# Improving the Flame Retardancy and Mechanical Properties of Vinyl Ester Resins through Maleated Epoxidized Corn Oil/Epoxy Resin Additives for Sustainable Thermoset Composites

**DOI:** 10.1021/acspolymersau.4c00088

**Published:** 2024-12-27

**Authors:** Maurelio Cabo, Prabhakar Manoj Narendra, Dong-Woo Lee, Ruiwen Yu, Vinitsa Chanthavong, Jung-Il Song

**Affiliations:** † Department of Smart Manufacturing Engineering, Changwon National University, Changwon 51140, Korea; ‡ Research Institute of Mechatronics, Department of Mechanical Engineering, Changwon National University, Changwon 51140, Korea; § Department of Mechanical Engineering, Changwon National University, Changwon 51140, Korea; ∥ Joint School of Nanoscience and Nanoengineering, University of North Carolina at Greensboro, Greensboro, North Carolina 27401, United States; ⊥ Bristol Composites Institute, School of Civil, Aerospace, and Design Engineering, Faculty of Science and Engineering, University of Bristol, Bristol BS8 1QU, U.K.

**Keywords:** epoxidized corn oil, epoxy resin, vinyl ester resin, thermoset blending, green composites

## Abstract

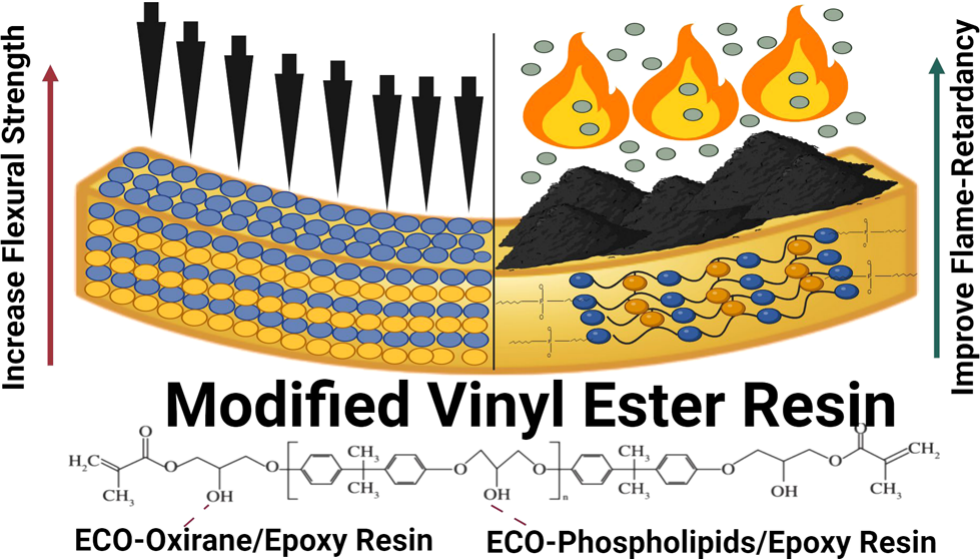

Thermoset polymers serve a significant role in modern industrial applications, and with a global annual output of over 65 million tons to meet this growing demand for sustainable materials, scientists and engineers need to go beyond what makes a material best for a certain use. Vinyl ester (VE) is a thermosetting polymer derived from polyester and epoxy resin. Its mixing properties distinguish it from its competitors, offering advantages in terms of curing efficiency, wettability, corrosion resistance, and low cost, which are crucial for modern industrial applications. Researchers have continuously explored the modifications of the intrinsic properties of VE using additives to enhance its flame retardancy and mechanical characteristics for more cost-effective and environmentally friendly materials applicable across various industries. In this study, we developed an easy-to-process eco-thermoset blend additive (50% v/v), known as maleated epoxidized corn oil/epoxy resin (MEPECO). Adding an optimal amount of MEPECO (5%) to the VE resin significantly improved its flame retardancy properties, as assessed by pyrolysis-combustion flow calorimetry, contact angle measurements, and thermogravimetric analysis. The mechanical properties, specifically strength, also showed substantial enhancement with the same optimal amount of MEPECO, as determined by flexural testing and spectral analysis. However, during the digestion of the eco-thermoset resin, the modulus and impact energy were notably lower owing to shear-yielding localization, as evidenced by the morphological analysis. This paper presents a novel in situ and straightforward technique for the easy and effective blending of eco-thermoset additives into petroleum-based epoxy resins, thereby facilitating their potential application in the development of sustainable green composite materials.

## 1 Introduction

Vinyl ester (VE) resins, a versatile class of thermosetting polymers, have found widespread use in protective coatings, chemical processing parts, construction materials, and polymer matrices for fiber-reinforced composites owing to their exceptional combination of mechanical strength, corrosion resistance, and ease of processing.^1−3^ However, their inherent brittleness significantly limits their industrial applications. To enhance the fracture toughness of VE resins, the incorporation of a secondary phase (rigid or flexible) is a widely acknowledged approach. The introduction of special additives enhances the positive characteristics of the polymer and, to some extent, diminishes its negative characteristics.^4^

Various fillers, including particulate, rubbery, fibrous, and resinous fillers, can effectively toughen the VE resins. Fillers act as the dispersed phase surrounded by the matrix phase and are thus solid additives introduced into a polymer to modify the physical characteristics essential for enhancing its applicability, such as its mechanical, thermal, and dynamic properties. Particularly, matrix resins utilize fillers for cost reduction, enhanced processing, density control, thermal conductivity, electrical characteristics, magnetic properties, mechanical properties, and flame retardancy, with a specific focus on the latter two, which are crucial for natural fibers or green composites.

Designing flame-retarding VE materials involves two main approaches: additive and reactive type.^5,6^ Currently, various phosphorus-containing additive flame retardants, including halogen, phosphorus, nitrogen, silicon, and metal oxide/hydroxide, are widely used;^7,8^ ammonium polyphosphate, 10-dihydro-9-oxo-10-phosphorus-phenanthene 10-oxide (DOPO), and their derivatives are few notable flame retardants.^9,10^

Yang et al.^11,12^ designed two types of intumescent flame retardants with high phosphorus and nitrogen contents, which remarkably enhanced toughness and flame-retarding efficiency. Another group from Yang et al. studied phosphaphenanthrene and triazinetrione groups as intrinsic sources of P–N-containing flame retardants, achieving improved VE resin flame retardancy through carbonization and a high concentration of free radicals.^13^ Zhu et al. experimented by dissolving liquid DOPO-containing 1-vinylimidazole salt in ethanol to improve flame retardancy in VE and its composites, showing a slightly higher storage modulus, slightly lower glass transition temperature (*T*_g_), and better mechanical performance compared with neat VE.^14^ Recently, exploration of using metal–organic framework to enhance flame retardancy of polymer elastomer like polyurea^15,16^ has provided new insights into the design of efficient biobased flame retardants to circumvent ineffective etching as well as the polymer composites. However, these additive techniques are limited by easy migration, poor compatibility, and interfacial forces between the flame retardants and the matrix.^17,18^ In contrast, reactive-type flame-retarding methods can overcome these limitations through copolymerization (olefin molecules with flame-retarding elements) and the designation of highly efficient curing agents.^19,20^ Nevertheless, this method faces challenges from any of the following: complex synthesis, low yield, and poor flame retardancy.

Additionally, adding phosphorus-based flame retardants to a resin typically results in a considerably lower *T*_g_, significantly lower initial weight loss temperature, and significantly worse mechanical performance of the cured resin.^21−23^ Another issue is phosphorus-induced eutrophication, which occurs when phosphorus enters water, and the phosphorus cycle must be addressed.^24^ Compounds are classified as high risk if they contain P, medium risk if they contain N, and low risk if they contain neither N nor P.^25^ P is an effective and widely used halogen-free ingredient in several commercial flame-retardant compounds.^26^ Thus, introducing nonphosphorylated epoxidized corn oil (ECO)^27^ as a thermoset resin with inherent flame-retardant properties as a promising filler to improve VE flame retardancy is the goal of this study.

Regarding the mechanical properties, the toughness of VE has been increased by blending it with an elastomer,^28^ a thermoplastic,^29^ or another thermoset like diglycidyl ether of bisphenol A-based epoxy resin^30^ and by incorporating inorganic nanoparticles, for instance, nanosized SiO_2_.^31^ Song et al.^32^ reported interpenetrating polymer networks using epoxy resin and polyurethane to enhance toughness, thermal stability, and mechanical strength. Tang et al.^33^ prepared a series of PU-based gradient IPNs from polyurethane/VE resin IPN systems that exhibited better mechanical and thermomechanical properties than those of conventional IPN. Parmar et al.^34^ reported that three-component IPN matrix-based glass-fiber-reinforced composites made with the VE of an epoxy novolac resin, diglycidyl ether of bisphenol A, and MMA exhibited resistance to chemicals and better thermal and mechanical properties compared with neat resin. Chen et al.^35^ fabricated polyurethane-based IPNs on TDI, graft polyol, and VE resin for the reactive injection molding process and found that the IPN prepared with graft polyol had the best mechanical properties among all specimens.

The use of thermoset-thermoplastic blends has gained much attention, owing to their enhanced mechanical properties and thermal stability. Thus, blending two polymers, which is an effective way to devise novel polymer materials with optimized properties, was attempted in this study to improve the toughness of VE. The main objective of this study was to explore the influence of a potential flame retardant and an additive that provided mechanical improvement, prepared from a recently reported thermoset blend of nonphosphorylated epoxidized vegetable oil (EVO) from corn oil and DGEBA epoxy resin.^36^ The synthesis of maleic anhydride via maleation followed by its addition to a VE polymer matrix was expected to enhance its flame retardancy and mechanical properties. To the best of our knowledge, this is the first time that a maleation process was introduced to easily blend epoxidized vegetable oil and epoxy resin additives into thermoset resins. However, environmental impact assessment was not included since our primary focus was to establish first the feasibility of synthesizing and characterizing the maleated epoxidized corn oil/epoxy resin (MEPECO)-based flame-retardant thermoset resin.

The optimal blend was further characterized by determining its properties, including flame retardancy (pyrolysis-combustion flow calorimetry, cone calorimetry, contact angle measurement, and thermal stability using a thermogravimetric analyzer (TGA)) and mechanical properties, (flexural, impact, and morphological analyses by scanning electron microscopy (SEM), optical microscopy (OM), and FTIR spectroscopy). The improvement in its flame retardancy and mechanical properties will serve as a clear reference for future studies on the use of nonphosphorylated epoxidized vegetable oil (EVO) combined with thermoset polymers to increase the structural green composite life cycle.

## 2 Experimental Section

### 2.1 Materials

The purchase of corn oil and maleic anhydride (99%) was made from Sigma-Aldrich (Korea). Acetone (99% volume/volume), glacial acetic acid (99.7% volume/volume), sulfuric acid (98% volume/volume), hydrogen peroxide (34.5% volume/volume), and diethyl ether (99.9% volume/volume) were acquired from Samchun (Korea). The EP (diglycidyl ether of bisphenol A-type resin with 184–190 g/EP equivalent and a density of 1.17 g/cm^3^) (YD-128) and methyltetrahydrophthalic anhydride (MTHA) (KBH-1089) were acquired from Kukdo Chemicals Co., located in Geumcheon-gu, Korea. The KRF-1031, which is a bisphenol A epoxy vinyl ester with a viscosity of 150 cps and a specific gravity of 1.03, was acquired from CCP Composites (Korea). Additionally, cobalt naphthalate was obtained as an accelerator, and methyl ethyl ketone peroxide was obtained as a hardener. The releasing agent utilized was Frekote 700-NC, specifically the component number 38425, made by Henkel. All of the resources utilized in this study were used in their original form without any modifications. The approaches were conducted in compliance with the applicable rules and legislation.

### 2.2 Synthesis of Epoxidized Corn Oil

**Figure 1:**
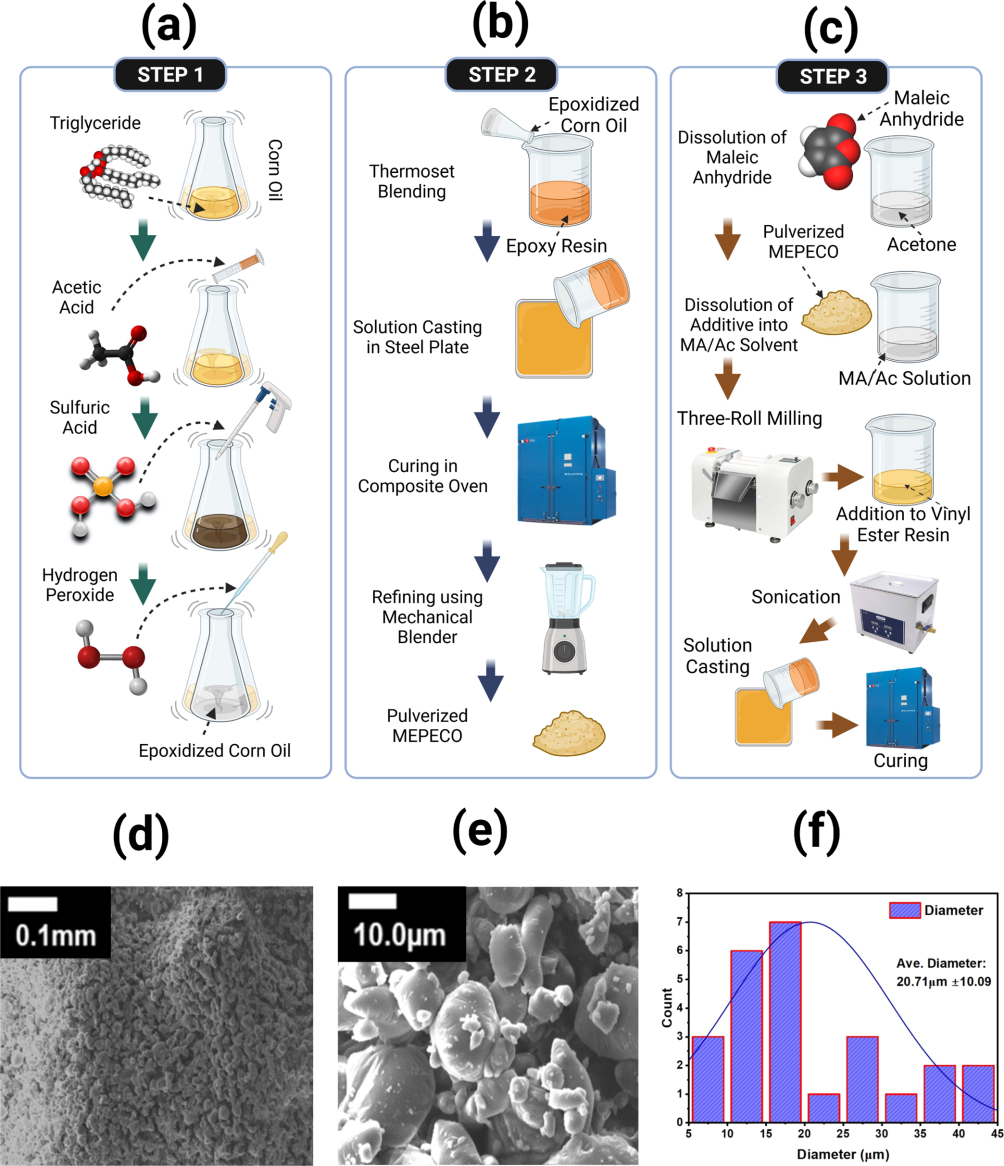
Schematic of the experimental procedures used in this study. First, the epoxidation of corn oil (a, step 1) is performed, followed by the fabrication and preparation of eco-thermoset additives through thermoset blending, curing, and mechanical blending (b, step 2). Finally, the maleation reaction is conducted using maleic anhydride (dissolved in acetone), which is simply added to vinyl ester resin (VER) through curing (c, step 3). SEM images of MEPECO at 0.1 mm (d) and 10 μm (e) are shown here measuring a particle size diameter of 20.71 μm ± 10.09 (f) on average.

A mixture of carboxylic acid and a liquid inorganic acid catalyst, comprising 1% of the total solution weight, was introduced into a vessel for the purpose of conducting a one-pot epoxidation reaction. The optimal reaction conditions were established by utilizing acetic acid and the necessary quantity of 34.5% aqueous H_2_O_2_ solution in the presence of a catalytic quantity of sulfuric acid. During a duration of 30 min, a suitable quantity of H_2_O_2_ was supplied gradually, one drop at a time. The duration of the synthesis was prolonged to 4 h. The synthesized substance was isolated by employing a separating funnel with diethyl ether as the solvent. The samples underwent a washing process using both cold and hot water to eliminate the presence of unbound acids. The corn oil epoxidation process was carried out under ideal reaction circumstances, which included a temperature of 50 °C, a rotational speed of 1200 rpm, a molar ratio of 10:1 of H_2_O_2_ to double bonds, and a molar ratio of 0.90:1.0 of acetic acid to double bonds. These settings were based on the previous study (Figure 1a).^27^

### 2.3 Thermoset Blending, Pulverization, and Maleation of Epoxidized Corn Oil and Epoxy

According to the optimal blend obtained in our previous study^36^ for the addition of epoxidized corn oil (ECO) into epoxy (EP) resin, thereafter, abbreviated as EPECO, the two materials were manually mixed in the percentage of 50% v/v, with MTHA as a hardener (5:4 ratio) on a molding plate. The material was then cured in a curing oven at 120 °C for 10 h. Using a high-speed waring laboratory blender (Model 7010S 1L 2 Speed Blender w/ Timer and Stainless-Steel Container, 120 V 50/60 Hz) set at a 3 min timer and turned on “HI,” the cured resin blend was pulverized until it was totally refined. Using SEM and ImageJ, the particle size diameter was measured and found to be on an average size of 20.71 μm ± 10.09 (see Figure 1d–f). EPECO powder (5% weight of the total volume) was dissolved in a 100 mL solution of maleic anhydride and acetone (1:2) with stirring at room temperature. The solution was then set aside for the subsequent procedure (Figure 1b). The dissolution of EPECO into an acetone-maleic anhydride solution helps to disintegrate the intermolecular bonding of EPECO for easy blending into VE. Herein, the established endothermic solubility of maleic anhydride in acetone^37^ and the nonchemical resistance of the thermoset resin to acetone were utilized.^38^

### 2.4 Optimization of VE_MEPECO

Maleated epoxy resin/epoxidized corn oil (MEPECO) and VE resin were blended using an overhead stirrer for 4 h at ambient temperature; see Table 1 for details. Sample blends were run thrice in a 3-roll mill, which is one of the most successful techniques for dispersion in viscous matrices.^39^ This was assumed to avoid the agglomeration of additives in the VE resin. Methyl ethyl ketone peroxide (curing agent) and cobalt naphthalate (accelerator) were both added at 1% of a constant volume of VE (300 mL) and sonicated for 1 h. The steel plate mold, with dimensions of 250 × 250 × 10 mm^3^ (*L* × *W* × *H*), was initially cleaned, Teflon-coated, and sprayed with a releasing agent to prevent the blend from sticking to it after removal. The mixture, hereafter referred to as VE_MEPECO, was poured into the steel mold. Precuring was performed at room temperature for 24 h, and the mold was directly placed into the curing oven composites at a curing temperature of 80 °C for 4 h. Finally, postcuring was performed at 120 °C for 2 h (Figure 1c). The curing temperature parameters were a slight modification from the optimal conditions of VE resin and epoxidized corn oil (ECO) blend from our previous study and available technical data sheet from En Chan Chemical Industries Co. Ltd.^40,41^

**Table 1:** Mixing Ratio and Nomenclature of Sample Blends

				precuring		on-curing		postcuring	
sample	VE (mL)	MEPECO (% w/v)	blend nomenclature	(h)	(°C)	(h)	(°C)	(h)	(°C)
1	300	0	VE_Neat	24	ambient	4	80	2	120
2		1	aVE_1%MEPECO						
3		3	VE_3%MEPECO						
4		5	VE_5%MEPECO						
5		7	VE_7%MEPECO						

a Vinyl ester (VE) resin: number of maleated epoxy resin/epoxidized corn oil.

### 2.5 Characterization and Testing

The flame retardancy properties of the samples were examined using pyrolysis-combustion flow calorimetry (Model Number: SG-5300) test apparatus, the FAA micro calorimeter (Federal Aviation Administration, Fire Testing Technology (FTT), U.K.), to calculate the heat release rate (HRR) according to ASTM D 7309 standards. It is a low-cost tool for screening and predicting the flame retardancy of polymers and other materials. In this flame retardancy test technique, during pyrolysis, the gases are released into an oven at 900 °C containing an 80:20 mixture of N_2_/O_2_.

According to ASTM E1354, the cone calorimeter test was also utilized to assess flame retardancy in terms of the HRR, carbon monoxide production (COP), and smoke production rate (SPR). Aluminum foil was used to cover the bottom side of the specimens with a size of 100 × 100 × 3 mm^3^, and they were horizontally subjected to a heat flux of 50 kW/m^2^.

The contact angles of the vinyl ester resin reinforced by maleated epoxy resin and epoxidized corn oil were measured with respect to a 10 μL deionized water droplet at room temperature by using a Phoenix Smart optical contact meter (SEO Co. Ltd., Korea). The reported contact angles are the average of three measurements made at different points on each sample and taken 10 ± 2 s after droplet placement.

A thermogravimetric analyzer (TGA; PerkinElmer STA 6000, England) was used to test the samples’ thermal stability across a temperature range of 30–700 °C and at a rate of 20 °C/min under a nitrogen atmosphere. The kinetic parameters were determined using a modified form of the Coats and Redfern model as described^42^ in the following equation
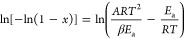
1where *x* is the first rate if reaction, *A* is the pre-exponential factor, β is the heating rate (20 °C/min), *R* is the general gas constant (8.3143 Jmol^–1^ K^–1^), *E*_a_ is the activation energy, and *T* is the temperature (K). Plotting graphs between ln[−ln(1 – *x*)] vs 1000/*T* for each phase gave the value of activation energy, and further parameters were determined using basic thermodynamic equations^43^
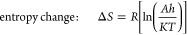
2where *h* is the Planck constant and *K* is the Boltzmann constant.
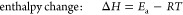
3
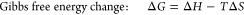
4

To study the surface morphology, the specimens were tested under an optical microscope (OM; Olympus, U MSSP4 model with Tech Xcam-III, Techsan Company Limited, Japan) and a scanning electron microscope (SEM) at 20 kV (Model: Emcrafs cube 2, EMCRAFTS.CO, Korea). The specimens were sputter-coated with gold using an autofine coater (JEOL JFC1600). Particle size average diameters were measured using ImageJ software (U.S. National Institutes of Health, Bethesda, Maryland), 25 times per sample.

The flexural strength was determined using an ASTM D790 standard on universal testing equipment (R&B Unitech, Korea) with a load cell of 50 kN. The specimens were sliced to a width of 12.7 mm with a span length of 16 times the thickness of the specimen, and the crosshead speed was kept constant at 1 mm/min. The sample cutting machine used here was manufactured by Korean Composites Application (KCA) with model number BK1500. The impact test was performed according to ASTM D256 standards using an Izod impact testing machine (model QC-639 °F, Cometech, Korea) with a capacity of 22 J.

To elucidate the interaction between VE and MEPECO along with the curing agents, spectral analysis from ATR-FTIR under dry air at ambient temperature was used. The IR spectra were recorded on a JASCO instrument (FT-IR-6300, FTIR spectrometer, U.K.). The percentage of transmittance spectra was collected with 32 scans at a resolution of 4 cm^–1^ in the 400–4000 cm^–1^ range. Furthermore, for the purity analysis in terms of epoxy groups content, the methods described by Zlatkovic et al.^44,45^ allowed the calculations of the concentration of the epoxy groups. Here, for each IR spectrum, the content of the epoxy group is determined by IR using the area of the peak at 3056 cm^–1^. The content of the epoxy group is determined by using the following equation^45^
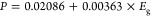
5where *P* is the IR peak area of absorbance at 3056 cm^–1^ and *E*_g_ corresponds to the epoxy groups content after a certain cross-linking time.

## 3 Results and Discussion

### 3.1 Effects on the Flame Retardancy of Vinyl Ester Resin

**Figure 2:**
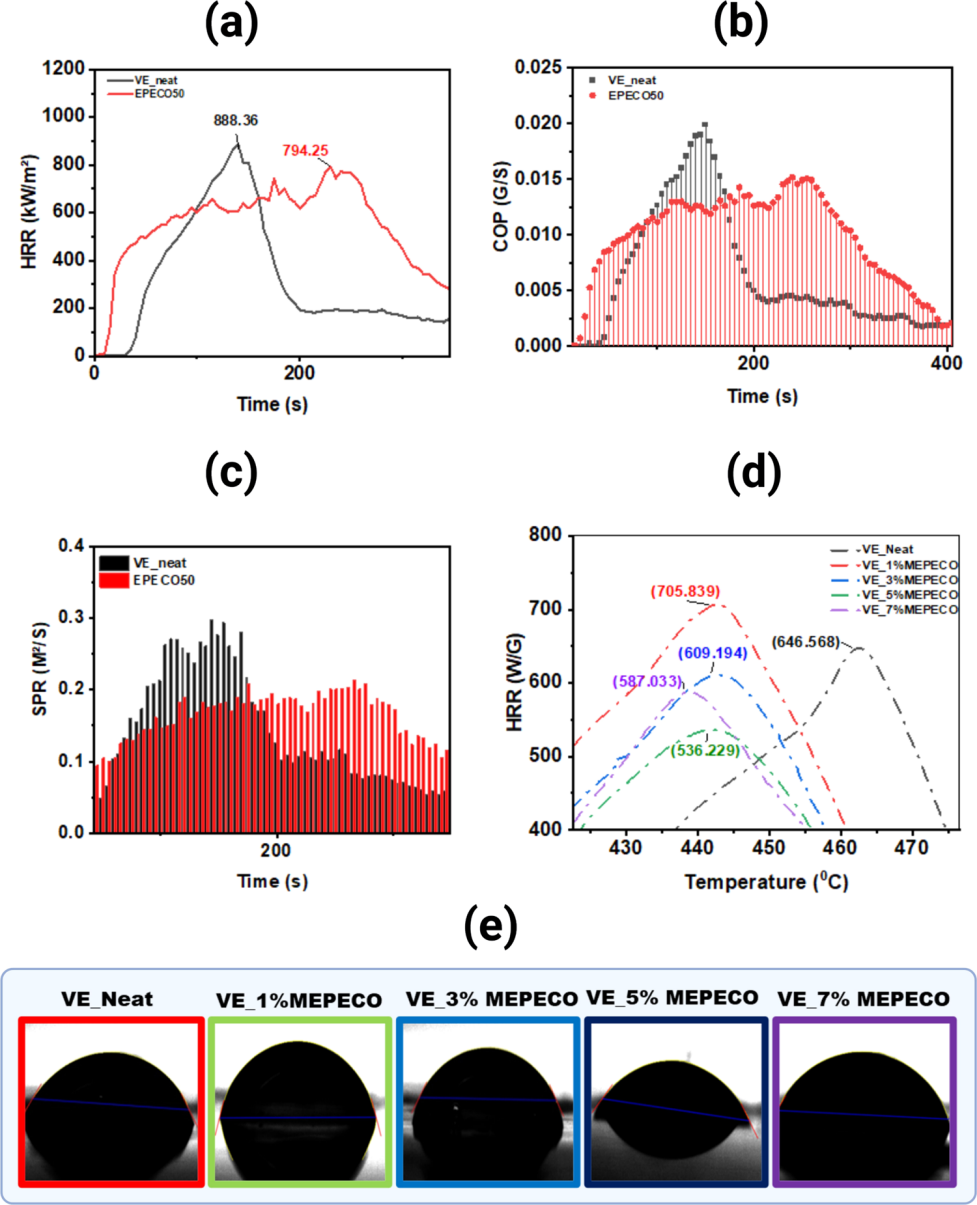
Comparison of the HRR (a), COP (b), and SPR (c) of EPECO original blend of 50/50 v/v% in cured form with those of pure epoxy resin and neat vinyl ester resin using a cone calorimeter. Optimization of the addition of the pulverized form of maleated-EPECO (MEPECO), to vinyl ester resin, was performed by analyzing HRR using PCFC or microcalorimeter (d) and contact angle (e).

To revalidate the flame retardancy of the cured thermoset blend obtained from DGEBA epoxy resin and epoxidized corn oil (EPECO50), parameters such as HRR, COP, and SPR were selected, evaluated, and analyzed to reveal the potential of the thermoset blend (EPECO50) to reduce the toxicity and fire hazards of VE resin. As shown in Figure 2a–c, the HRR, COP, and SPR values for EPECO50 are 794 kW/m^2^, 1.45 × 10^–2^ G/S (COP), and 18.08 × 10^–2^ M^2^/S, respectively. For the VE resin, the values are 888.36 kW/m^2^, 1.89 × 10^–2^ G/S, and 26.12 × 10^–2^ M^2^/S, respectively. Compared with the VE resin, EPECO50 demonstrates a 10.59% lower HRR, 23.13% reduced COP, and 30.77% improvement in SPR.

HRR is one of the most important parameters for describing the process of fire; it determines the energy release in a fire and is a basic parameter of the degree of fire danger.^46^ The peak heat release rate represents the maximum heat release rate of a material.^3,47^ The average heat release rate represents the average level of heat released during a fire event. The higher the average heat release rate, the more violent material burns.^13^ The significance of lowering the SPR can be analyzed as EPECO50 demonstrates that it is more effective in reducing smoke at a high incident heat influx. The results also indicate that the introduction of epoxidized corn oil into the DGEBA epoxy resin in the form of an interpenetrating network reduces smoke generation in the blend. In addition, CO is the most common toxic constituent of smoke in building fires. Therefore, CO generation was used to measure the fire risk rate of important material parameters. The significantly lower value of EPECO50 compared with that of the VE resin might be due to the absence of high-temperature fracture and combustion of the polymer chain.

Based on our previous study, and as shown in the graphical abstract, we proposed that the improvement was due to the catalytic effect of P-based fragments from phospholipids and O–H radicals.^36,40^ Meaning, the interaction between ECO-phospholipids and epoxy resin (EP) caused the flame retardant and highly reduced toxicity properties. The flame-retardant behavior interacted with the VE resin, increasing the carbon content on the matrix. These groups exhibited inherent flame-retardant effects and retarded heat release from the DGEBA epoxy matrix during combustion. In a fire incident, victims die due to the lack of oxygen and inhalation of toxic smoke; therefore, the reduction in COP and SPR indicates the smoke suppression effect of epoxy thermosets.^48^

**Figure 3:**
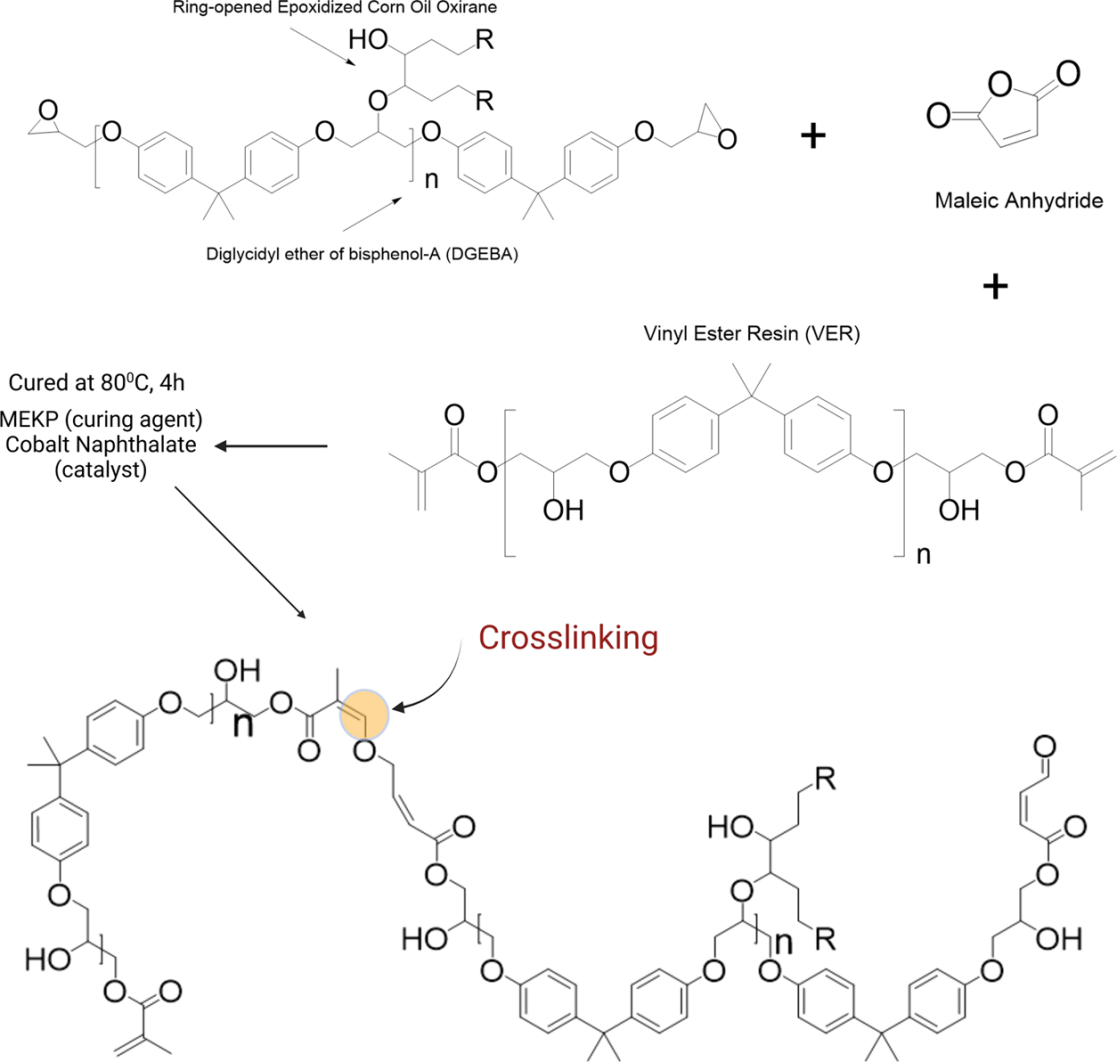
Proposed mechanism between maleic anhydride, MEPECO, and vinyl ester resin upon curing.

The maleation process, wherein maleic anhydride was dissolved in acetone to solubilize EPECO50, was chosen to determine the influence of EPECO50 in terms of flame retardancy because of its capacity to perform hydrolysis and cleave chemical bonds.^49^ In hydrolysis, because acetone contains a carbonyl (C=O) group and maleic anhydride contains an anhydride functional group, a water molecule can be released upon interaction with the thermoset polymer. We hypothesized that hydrolysis would increase the hydrophilicity of the blend. Also, we can look at the proposed mechanism as follows: the acetone-maleic anhydride solution facilitates blending EPECO into vinyl ester resin (VER) by acting as both a solvent and a compatibilizer. Acetone dissolves maleic anhydride and EPECO, reducing viscosity for uniform dispersion, and evaporates during curing, leaving no residue. Maleic anhydride reacts with EPECO’s epoxide groups, forming maleated EPECO (MEPECO), which enhances compatibility with VER by promoting chemical cross-linking during curing (see Figure 3).

After maleation with maleic anhydride, addition to VE resin was optimized using loading amounts of 0, 1, 3, 5, and 7%. Figure 2d shows the optimization of the amount of MEPECO using a PCFC by obtaining the HRR value. Herein, 5% MEPECO added to the VE resin (VE_5%MEPECO) showed a 17.07% increase in the HRR value (536.229 kW/m^2^). The remaining trends in PCFC HRR are as follows: VE_7%MEPECO (587.033 kW/m^2^) > VE_3%MEPECO (609.194 kW/m^2^) > VE_Neat (646.568 kW/m^2^) > VE_1%MEPECO (705.839 kW/m^2^).

**Figure 4:**
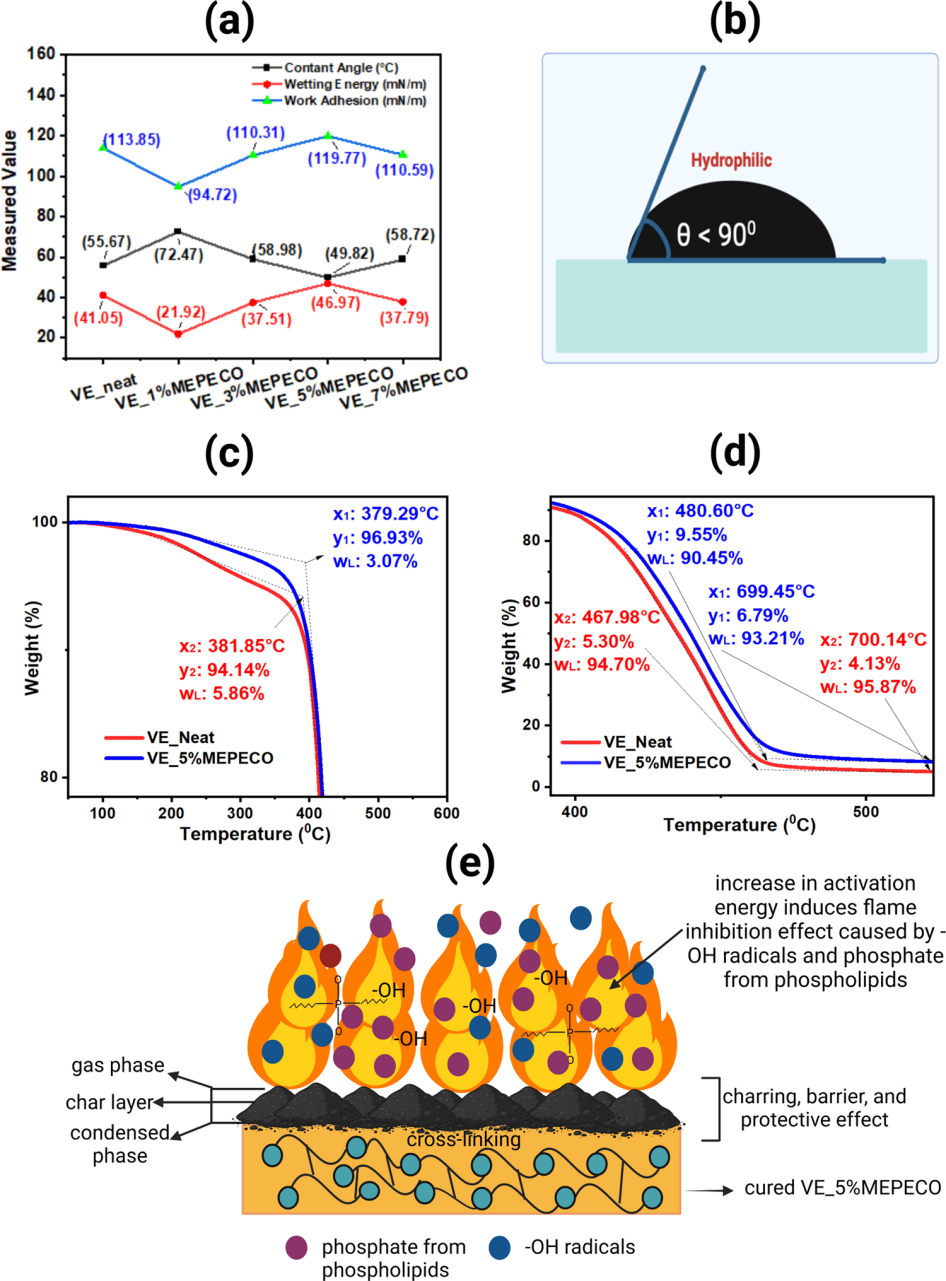
Flame retardancy investigation: (a) Wettability analysis via contact angle, wetting energy, and work of adhesion. (b) Schematic showing that the material is hydrophilic for contact angles less than 90° contact. Thermogravimetric analysis of the VE_Neat and VE_MEPECO blends was used to determine their thermal stability. The changes are centered on the following: (c) first degradation or dehydration; (d) second degradation or decomposition; remaining yield or condensation. (e) The proposed flame-retardant mechanism of VE_5%MEPECO.

For the contact angle, as shown in Figures 2e and 4a, VE_5%MEPECO exhibits a 10.51% (49.82°) improvement over VE_Neat (55.67°). Hence, the flame retardancy and contact angles both confirm that the addition of 5% MEPECO improves the hydrophilicity (contact angle less than 90°) of the VE resin, which tends to absorb large amounts of water molecules, which is consistent with our hypothesis. Surface hydrophilicity is an important paradigm for eliminating microbial colonization. Studies have shown that a surface hydration layer induced by hydrophilic polymers may impart antibiofouling properties.^50^

Herein, modifying the interfacial interactions between the resin matrix and the reinforcing agents or additives can improve the dispersion and compatibility of additives, ultimately influencing the resin’s performance characteristics; for instance, the polar groups in maleated epoxidized corn oil can form hydrogen bonds or interact with polar filler surfaces, leading to better distribution and interfacial adhesion. This uniform dispersion improves the flame-retardant efficiency by creating a homogeneous thermal barrier during combustion, reducing heat transfer and material degradation.

In addition, the surface interaction of water molecules with the surface of the VE resin produced by the hydroxyl radicals from the MEPECO additive forms an additional layer of tightly bound water, which functions as an energetic and physical barrier that effectively resists the biofouling process. Furthermore, wettability, the ability of a liquid to maintain contact with a solid surface, was investigated by determining the wetting energy and work of adhesion. As shown in Figure 4a, VE_5%MEPECO requires higher wetting energy and exhibits higher work of adhesion of 46.97 and 119.77 mN/m, respectively. Typically, at a high wetting energy, the liquid spreads completely, lowering its surface energy and increasing its interfacial attraction.^51^ Consequently, an increase in interfacial attraction results in an increase in the work of adhesion.^52^ These two parameters also confirm that 5% MEPECO is the optimal amount to be added to the VE resin to improve its flame retardancy.

Table 2 shows the values of additional parameters, such as the height from top to base, baseline length, and base area. The parameter values for VE_5%MEPECO are the highest among the other samples. Although only 4.25 volumes of water were dropped onto its surface, the expected hydrophilic phenomenon (Figure 4b) was observed using VE_5%MEPECO. To correlate the importance of thermal stability, we further analyzed the thermodynamic kinetics between VE_Neat and the optimal VE_5%MEPECO by TGA. The first step (Figure 4c) and second step (Figure 4d) degradations were determined as the temperature at the intersection points: the tangent line to the start of decomposition and the tangent line to the maximum weight loss rate. The MEPECO additive caused a shift in the TGA graph toward the right. This finding indicates that compared with pure VE resin, a higher temperature is required to initiate devolatilization of the samples for additives. A possible chemical reaction occurred between the two polymeric components during mechanical blending. As a result of this process, a chemical reaction occurred within the interphase; hence, an interfacial agent was produced in situ, increasing its thermal stability.^53^

**Table 2:** Additional Parameters for Contact Angle to Measure Hydrophilicity

parameters	unit	VE_Neat	VE_1% MEPECO	VE_3% MEPECO	VE_5% MEPECO	VE_7% MEPECO
height from top to base	mm	1.01	1.24	1.06	0.94	1.04
base line length	mm	3.89	3.51	3.8	4.04	3.84
base area	mm^2^	11.89	9.65	11.37	12.81	11.57
drop volume	μL	5.64	7.29	6.53	4.25	5.11
recorded temperature	°C	20	20	20	20	20

Furthermore, considering the three-phase transformations as first-order reactions, the activation energy and other thermodynamic parameters were analyzed using the first-order reaction eqs 1 and 4. Each phase follows a straight line, confirming that the transformations are first-order reactions. The slope of each line, which is equal to the value of the rate constant (*k*), can be determined as follows^42^
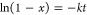
6Where
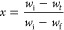
7where *w*_i_ is the initial weight, *w_t_* is the weight of the sample at time *t*, *w*_f_ is the final weight, and *t* is the half-life (*t*_1/2_) of the reaction, which is the time at which the concentration of the reactant is reduced to half of its initial concentration.^54^ The values of *k*, *t*_1/2_, activation energy (*E*_a_), change in entropy (Δ*S*), change in enthalpy (Δ*H*), and change in Gibbs free energy (Δ*G*) are listed in Table 3.

**Table 3:** Kinetic and Thermodynamic Parameters of Each Phase during the Thermogravimetric Analysis of VE_Neat and VE_5%MEPECO

	*T* (K)	*k* (min^–1^)	*t*_1/2_ (min)	*E*_a_ (Jmol^–1^ (×10^4^))	Δ*S* (Jmol^–1^ K^–1^)	Δ*H* (Jmol^–1^ (×10^4^))	Δ*G* (Jmol^–1^ (×10^5^))
First Degradation							
VE_Neat	652.29	3.06	0.23	2.545	–282.95	2.002	2.0459
VE_5%MEPECO	654.85	4.03	0.17	3.353	–270.30	2.808	2.0509
Second Degradation							
VE_Neat	740.98	22.52	0.0308	1.872	–26.03	1.811	2.003
VE_5%MEPECO	753.60	22.50	0.0308	1.871	–28.31	1.808	2.021
Residue Yield							
VE_Neat	973.14	2.83	0.303	1.899	–277.61	1.09	2.811
VE_5%MEPECO	972.45	2.48	0.279	2.061	–275.64	1.25	2.806
Average							
VE_Neat		9.47	0.19	2.11	–195.53	1.55	2.287
VE_5%MEPECO		9.67	0.16	2.43	–191.42	1.99	2.293

In the first degradation, the extrapolated onset temperature (*T*_0_),^38^ which denotes the temperature at which the weight loss begins and is specified to be used by ASTM 2550, is measured.^55^ The addition of 5% MEPECO improves the thermal stability from 652.29 K for VE_Neat to 654.85 K for VE_5%MEPECO, at weight loss. The addition of MEPECO improves thermal stability by losing considerably less weight compared with VE_Neat. The higher slope (*k*) for VE_5%MEPECO (4.03 min^–1^) than that of VE_Neat (3.06 min^–1^) also confirms the above result. The *t*_1/2_ values confirm that even though the concentration of the samples reduces shortly from 0.17 to 0.23 min upon the addition of 5% MEPECO to VE, because of the presence of less moisture and the aliphatic compounds, cross-linking and filling of 5% MEPECO into VE are controlled, and the inherent flame-retardant property of 5% MEPECO induces a considerably lower weight loss at the onset temperature.

The increased activation energy induced by VE_5%MEPECO (3.353 × 10^4^ Jmol^–1^) and VE_Neat (2.545 × 10^4^ Jmol^–1^) proves that the addition of MEPECO results in improved thermal stability, implying that more energy is required to degrade the inter- and intramolecular bonding of VE upon heat exposure. The entropy of VE_5%MEPECO (−270.30 Jmol^–1^ K^–1^) is significantly higher than that of VE_Neat (−282.95 Jmol^–1^ K^–1^), demonstrating that the presence of MEPECO triggers a faster catalyst that enchains many monomers in a macromolecule and induces the formation of the interactions between the terminal groups of VE resin and MEPECO. The presence of biopolymers in epoxidized corn oil makes the thermal equilibrium not constant, thereby creating more disorder.^38^ In addition, the long chain structure of DGEBA, with the presence of aromatic compounds forming the entire structure upon application of heat (curing), makes the polymer jiggly, increasing the entropic contribution.

Moreover, the enthalpy and Gibbs free energy are significantly higher than those of VE_Neat. In terms of bond energies, wherein hydroxyl radicals (O–H), 467 kJ/mol, C–O, 358 kJ/mol, C–C, 347 kJ/mol, from oxirane of epoxidized corn oil and additional C=C, 614 kJ/mol, and C–H, 413 kJ/mol,^56^ from epoxy resin, its further introductions to VE resin produce an exchange of Π-bonds from the double bonds and σ-bonds from the single bonds, creating a negative Δ*H*. Thus, the more exchanges of Π*-* and σ-bonds, the higher the enthalpic changes.^57^ A positive Gibbs free energy value indicates nonspontaneous reactions of the samples, which are attributed to the need for an external force such as heat to induce reactions, resulting in the final product.

The second degradation rapidly follows the first degradation and almost shows the same trend as the first degradation, that is, an increase in thermal stability and Gibbs free energy. However, unequal dispersion may be expected because of the presence of the catalyst, cobalt naphthalate, and cross-linker, MEKP, which were added manually. The slope of the line (*k*), activation energy, Δ*S*, and Δ*H* are slightly higher for VE_Neat, compared with VE_5%MEPECO. In contrast, residue yield shows slightly lower thermal stability, slope of the line, *t*_1/2_, and Gibbs free energy of VE_5%MEPECO compared with VE_Neat but slightly higher activation energy, Δ*S*, and Δ*H*.

Considering the average values of *k*, *t*_1/2_, *E*_a_, Δ*S*, Δ*H*, and Δ*G*, generally, VE_5%MEPECO shows an improvement in thermodynamic kinetic parameters (15.16% increase in activation energy). We can further assess that the correlation between the thermal stability and flame retardancy of thermoset resin blends is an important consideration when designing materials for various applications. Understanding the relationship between thermal stability and flame retardancy is crucial to ensure the safety and performance of polymer matrix (resin) materials. In many cases, there is a trade-off between thermal stability and flame retardancy. However, in our study, the potential improvements in both flame retardancy and thermal stability of VE resin are an important result because materials must withstand high temperatures and be fire-resistant, for instance, in developing structural composites called empennage side panels for the aerospace industry.^58^ In automotive and electronic industries, where heat dissipation is important, thermoset resin blends with good thermal stability are used, and their flame-retardant properties are crucial for preventing fires.^47^

Thus, the MEPECO enhances the compatibility with vinyl ester resin (VER) by promoting cross-linking through maleic anhydride groups, improving interfacial adhesion leading to improved flame retardancy and thermal stability (see the proposed flame-retardant mechanism in Figure 4e).

### 3.2 Effects on the Mechanical Properties of Vinyl Ester Resin

**Figure 5:**
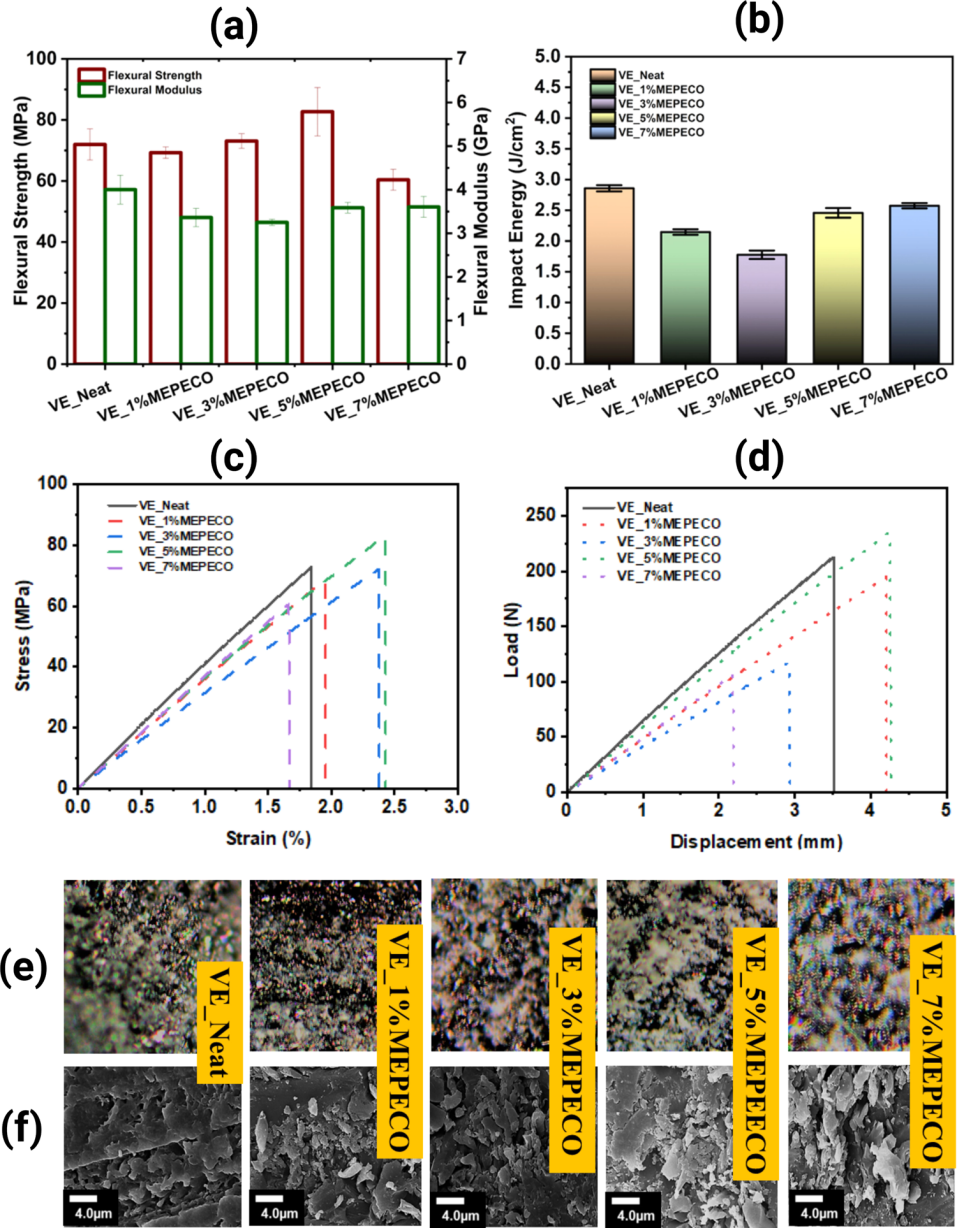
Measurements of the mechanical properties of VE_MEPECO blends: flexural strength and modulus (a), impact test (b), stress vs strain (c), and load vs displacement (d). Optical microscopy (10× resolution) (e) and SEM images (f) of the samples with increasing MEPECO content. The gradual increase in MEPECO distribution allows it to cover the microstructural defects of VE resin, like voids and microcracks, improving its mechanical properties.

To study the effect of the addition of MEPECO to VE in terms of mechanical properties, the changes in the flexural strength and flexural modulus of the VE resin were evaluated upon the addition of MEPECO at different loading percentages (0, 1, 3, 5, and 7%). Figure 5a demonstrates and agrees with the flame retardancy analysis result that the optimal additive loading is at 5% MEPECO. The flexural strength of VE_5%MEPECO is greater than that of VE by approximately 14.93% (82.68 MPa). The increase in flexural strength may be due to the effective reinforcing agent; at 5%, it enhances the structural integrity of the resin matrix. The possibility of homogeneous dispersion and distribution of 5% MEPECO over the VE resin molecules to fill the voids and microcracks may produce an effective filler–matrix interaction, leading to improved strength. Another possibility is that the addition of 5% MEPECO improves the cross-linking density of the epoxy resin and causes changes in the VE resin, regardless of the phase structure. However, the apparent decrease in the flexural modulus of approximately 10.39% (3.586 GPa) may be due to the uncontrollably lower elastic modulus of 5% MEPECO, which can lead to a decrease in the overall stiffness of the composite. Figure 5c,d supports this result, as VE_5%MEPECO tends to withhold a higher stress and load capacity, resulting in a higher flexural strength.

VE_3%MEPECO also shows a slight increase yet an apparent decrease in the elastic modulus. VE_1%MEPECO and VE_7%MEPECO exhibit significantly low strength and modulus, possibly because 1% is too low for the additive to fill the microstructural defects between VE molecules and 7% is too high, which leads to agglomeration or clustering of the additives over the surface and between the VE molecular structures. A possible reduction in the rigidity and internal friction of the VE network results in a significantly lower strength and modulus.

Although the flexural strength improves for 5% MEPECO, the impact test results follow the opposite trend. Figure 5b shows that all loading percentages induce a lower impact energy. Thus, the ability of VE to absorb energy is reduced by the introduction of the additive. Likewise, microstructural changes might also create a more rigid and less ductile environment, leading to a lower impact energy. Another assumption is that the adhesion between the VE and MEPECO molecules is not strong enough to resist the impact. It can also be assumed that the improved hydrophilicity of the blend leads to the formation of weak interfaces, resulting in lower energy absorption.

To further investigate the effect of the distribution of MEPECO on the mechanical properties of VE, morphological analysis was performed by optical microscopy (OM) and scanning electron microscopy (SEM). Figure 5e shows OM images, which provide valuable insights into the distribution and dispersion of MEPECO on the surface of the VE resin. The dispersion of a much wider whitish surface with the gradual increase of the MEPECO content imply aggregation and phase separation within the VE resin. This also suggests that a change in the optical properties or refractive index confirms the presence of MEPECO in the VE resin. The gradual increase in the area occupied by the white surfaces may indicate that the maleated epoxy resin is not homogeneously distributed within the VE resin. Instead, it may form localized clusters or domains.

The nonuniform distribution of additives can have a significant impact on the mechanical, thermal, and chemical properties of the composite. An uneven distribution can result in variations in the material strength, stiffness, and resistance upon the application of an external force. Furthermore, as shown in Figure 5f, the SEM images show that exposure to the curing process produces microstructural defects such as voids, cracks, and homogeneities in the polymer matrix. These defects weaken the material and degrade the material’s overall performance. SEM images reveal the interaction of MEPECO with these defects. The coverage of the microstructural defects by MEPECO depends on its chemical properties, viscosity, and processing conditions. As shown in the SEM images, MEPECO successfully penetrates these defects and forms a protective barrier. Moreover, in VE_5%MEPECO, the effective coverage of microstructural defects improves the mechanical properties of the composite material. It also enhances the strength and toughness by reducing stress concentrations at defect sites, preventing anticipated crack propagation, and improving load distribution. SEM analysis may also reveal instances of incomplete coverage, where MEPECO does not adequately address certain defects. This can be due to poor mixing, insufficient curing, or incompatibility between the indicated number of additives and the resin.

**Figure 6:**
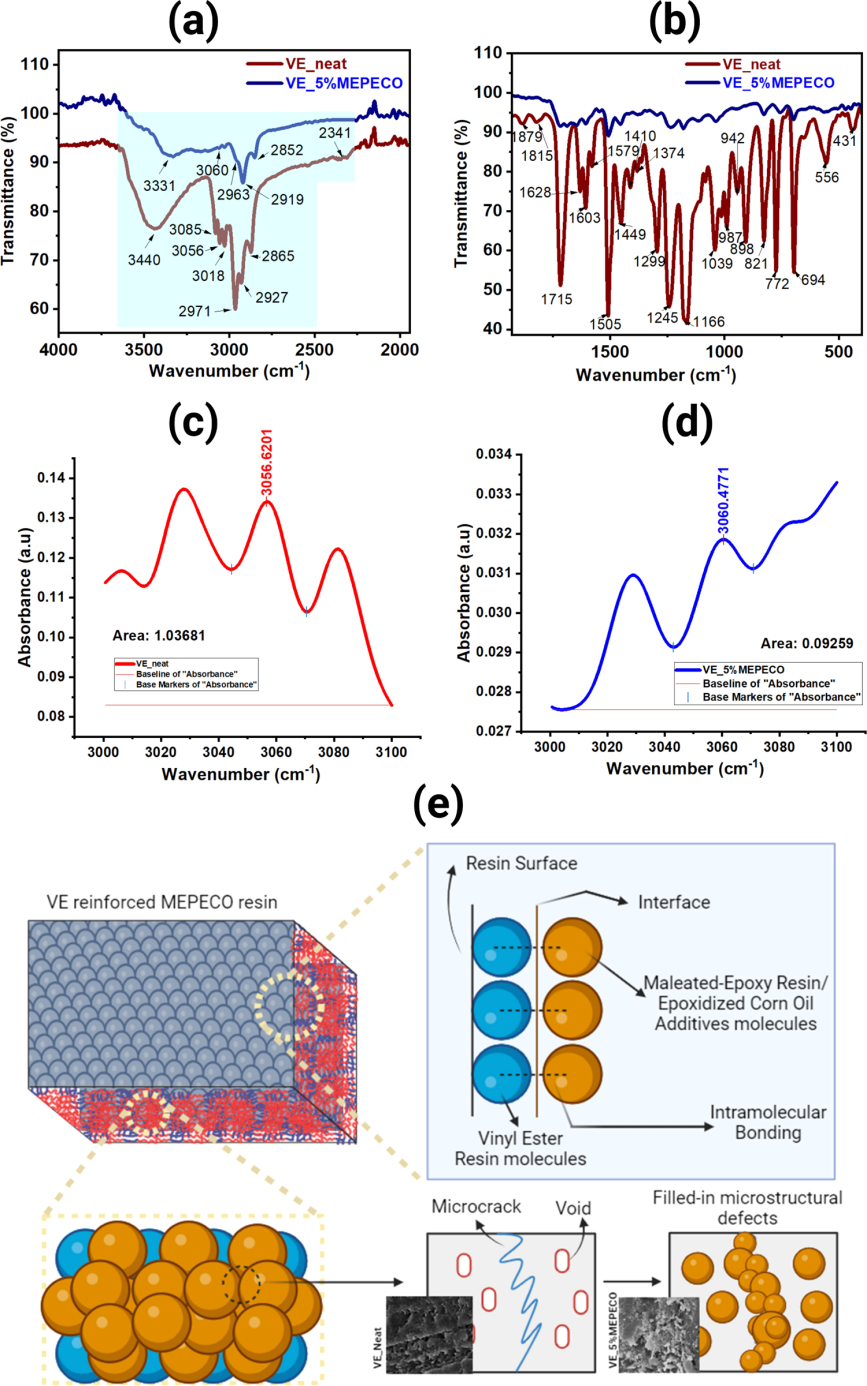
FTIR spectra of VE_Neat and VE_5% MEPECO in the fingerprint (a) and functional group (b) regions. The peak areas at 3056 cm^–1^ for VE_neat (c) and 3060 cm^–1^ (d) for VE_5%MEPECO were integrated to estimate the epoxy group content. Proposed mechanism (e) showing that the microstructural defects of vinyl ester resin are filled by MEPECO to cover the microcracks and voids and act as an “adhesive” between vinyl ester molecules, where the defects are formed after curing.

In addition, we examined the changes in the intermolecular structure of the VE resin by FTIR analysis to determine why the mechanical properties improved at the optimal amount of MEPECO (5%). Figure 6a,b shows the FTIR spectra of the functional group region and the fingerprint region, respectively. Significant reduction in the peak intensities of the pure VE resin is observed upon the introduction of 5% MEPECO. This reduced intensity may indicate that MEPECO reacts with VE resin, suggesting the formation of new cross-links and chemical interactions. This also confirms the improved adhesion, as VE_5%MEPECO previously showed wettability properties, which mainly improves the mechanical properties in terms of flexural strength and decreases the flexural modulus. The possible homogeneity and dispersion of 5% MEPECO over the VE resin might also be the reason for the overall changes in the peak intensities of VE_neat. FTIR designation of the peaks is as follows: 3331 and 3440 cm^–1^, −OH stretching; 3018, 3056, 3060, and 3085 cm^–1^, sp^2^ C–H stretching; 2341, 2852, 2865, 2919, 2927, and 2971 cm^–1^, sp^3^ C–H stretching; 1603, 1628, 1715, 1815, and 1879 cm^–1^, C=O stretching; 1505 and 1579 cm^–1^, aromatic C=C vibrations; 1410 and 1449 cm^–1^, O–H stretching, 1374 cm^–1^, bending; 1245, 1299, 1039, and 1166 cm^–1^, C–O stretching; 987, 942, 772, 694, 556, and 431 cm^–1^, C=C bending; 898 and 821 cm^–1^, C–H bending.^59^

Likewise, we determined the purity of the samples by calculating the epoxy group content. Figure 6c shows that at 3056 cm^–1^, pure VE resin has an integrated area of 1.03681; using eq 5, this translates to *E*_g_ at 279.88 mg/2 g, while in Figure 6d, VE_5% MEPECO shows an obvious shift of peak to 3060 cm^–1^ with an integrated area of 0.09259 translating into *E*_g_ at 19.76 mg/2 g (see Table 4 for consolidation of data). This decrease shows that the addition of MEPECO into VE_resin confirmed the high cross-linking activity and a faster conversion of epoxy groups that reflect a more effective curing process, which minimizes residual monomers or unreacted groups.^60^ In addition, the decrease in the transmittance at 821 cm^–1^, which is designated for the oxirane band, confirmed this high conversion of epoxy; however, interestingly, compared to our previous study,^36^ and as shown in the graphical abstract, the interaction between ECO-oxirane did not yield a lower mechanical strength for VE_5%MEPECO but instead offered enough increase in flexural strength. Figure 6e shows the proposed mechanism by which MEPECO allows the bridging of VE_neat molecules to cover the microstructural defects after curing. VE_Neat exhibits microcracks and voids filled by MEPECO, showing a homogeneous dispersion that helps to improve the strength and modulus. Thus, proper dispersion of the pulverized MEEPCO as facilitated by 3-roll mill technique ensures uniform interaction, leading to better mechanical properties. In addition, thermosets with higher thermal stability exhibit reduced thermal degradation, ensuring the preservation of structural integrity during processing or use at elevated temperatures.^61^

**Table 4:** Epoxy Group Content (*E*_g_) from IR Spectra

samples	peak assignment (cm^–1^)	peak area	*E*_g_ (mg/2 g)
VE_Neat	3056	1.03681	279.88
VE_5%MEPECO	3060	0.09259	19.76

## 4 Conclusions

In addition to addressing the challenge of investigating the flame retardancy and mechanical properties of polymers, in this study, we not only unveiled the potential of maleated epoxy resin/epoxidized corn oil thermoset blend, which provided a solution for the in situ processing of VE polymer networks, but also utilized the unique cross-linking effect of epoxidized corn oil into thermoset polymers to improve their properties. Thus, an efficient and environmentally friendly flame-retardant, MEPECO, added to VE resin was synthesized, optimized, and characterized.

Regarding flame retardancy, the experimental results confirmed that the addition of 5% MEPECO to VE resin increased the HRR value by 17.07%. This improvement was confirmed by the contact angle of 49.82° for 5% MEPECO, which was 10.51% greater than that of pure VE. TGA showed that a 15.16% increase in the activation energy maximized the thermodynamic capacity to become thermally stable.

In terms of mechanical properties, a significant upgrading of the flexural strength at 14.93% determined the capacity of MEPECO to allow the VE resin to withstand a significant amount of stress and load upon application of an external force. SEM images showed the dispersion of the additives over the microstructural defects of the VE after curing. FTIR spectral analysis also supported this claim regarding the overall reduction in the intensities of the functional groups, indicating the formation of new cross-links and chemical interactions. Overall, MEPECO is a novel and efficient eco-thermoset additive with potential value owing to its ability to improve the flame retardancy and mechanical properties of VE resins. Hence, it is a potentially attractive additive for the green composite and coating industries.

## Data Availability

Data are available in this article.
